# Effectiveness of Defocus Incorporated Multiple Segments in Slowing Myopia Progression in Pediatric Patients as a Function of Age: Three-Year Follow-Up

**DOI:** 10.3390/diseases12090222

**Published:** 2024-09-18

**Authors:** Luca Buzzonetti, Sergio Petroni, Matteo Federici, Paola Valente, Giancarlo Iarossi

**Affiliations:** Ophthalmology Unit, Bambino Gesù IRCCS Children’s Hospital, Via Torre di Palidoro snc, Passoscuro, 00050 Rome, Italy

**Keywords:** pediatric ophthalmology, myopia, defocus incorporated multiple segments lenses

## Abstract

**Background**: The purpose of this study is to evaluate the effectiveness of Defocus Incorporated Multiple Segments (DIMSs) in slowing myopia progression in pediatric patients as a function of age. **Methods**: This was a non-randomized experimenter-masked retrospective controlled observational study of European individuals aged 6–16 years with progressive myopia but no ocular pathology. We retrospectively reviewed the charts of the participants allocated to receive DIMS spectacles (Hoya^®^ MiyoSmart^®^) or single-vision spectacle lenses (control group). Cycloplegic spherical equivalent (SE) and axial length (AL) were measured at baseline and at 12-, 24-, and 36-month follow-ups. The results were stratified by age into four groups: patients wearing DIMS spectacles older or younger than 10 years of age (group A, 20 patients mean age 13.6 ± 2.2, and group C, 20 patients mean age 9.0 ± 1.2) and age-matched control groups (group B, 18 patients mean age 13.2 ± 2.5, and group D, 22 patients mean age 8.5 ± 0.9). **Results**: At 36 months, SE and AL increase were significantly reduced in groups A and C, respectively, compared to groups B and D (*p* < 0.05). Linear regression analysis showed a significant correlation (*p* < 0.05) between patient age and myopia progression for SE in groups A and C, but only in group A for AL. Groups B and D did not show any significant correlation (*p* > 0.05). **Conclusions**: DIMS spectacles seem to slow myopia progression in pediatric patients; however, their effectiveness shows the greatest results in children older than 10 years of age. Moreover, our findings suggest that AL may be the more reliable parameter for evaluating myopia progression.

## 1. Introduction

Myopia is a significant public health concern, affecting an increasing number of individuals worldwide [1]. Approximately 30% to 50% of adults in the USA and Europe have myopia [2]. The prevalence of myopia is increasing everywhere, and it is estimated that in 2050, 50% of the world population will be myopic [3].

The Correction of Myopia Evaluation Trial (COMET) study defined progressive myopia for children aged 6 to 12 years as refractive error progressing more than −0.75 diopters (Ds) per year. However, in clinical practice, patients newly diagnosed with myopia often only have the refractive error measurement available at a single point; previous data with well-documented myopia progression over time are not present. In addition, a single cut-off of spherical equivalent to define progressive myopia for children aged 5–18 years may not be accurate [4]. Previous studies indicated that the mean annual myopia progression rate in children was about half-a-diopter in Europe (−0.55 Ds) and slightly higher in Asia (−0.82 Ds) [5].

Vision-threatening complications are frequently correlated with high myopia, with an associated elevated risk of ocular diseases such as retinal detachment, glaucoma, and myopic maculopathy, which is one of the leading causes of low vision and blindness in developed countries [1,6,7,8].

Few prospective studies have shown the relationship between age at myopia onset and myopia severity [9,10,11], but since the high prevalence of myopia provides important public health and socio-economic problems [12], and because the rising prevalence of myopia mainly occurs in the youngest population, the need to identify more effective interventions for managing myopia progression is growing. In this effort, evaluating the effectiveness of each possible treatment as a function of age could assume a special importance [1,13,14]. Because myopia is usually detected in children before 10 years of age and the prevalence of myopia can progress quickly after the age of six, and although the sight-threatening pathologies associated with myopia usually occur later in life, the therapeutic strategy to slow myopia increase needs to be provided in childhood in an attempt to reduce a subsequent visual impairment [15]. 

Several different therapeutic options are considered for slowing myopia progression [16]. Pharmacological treatment proves relatively more effective than optical methods, such as wearing contact lenses or spectacles [17,18]. Lower-dose (0.01–0.1%) atropine is frequently prescribed because it induces fewer associated side effects, such as photophobia and blurry vision, and some clinical trials indicated that 0.01% concentration atropine eyedrops achieve modest therapeutic effects but low myopic rebound and minimal side effects [19,20,21,22].

Theoretically, the preferred treatment should be as non-invasive as possible, consequently making optical devices the ideal alternative option [16]. Optical interventions include a variety of spectacle and contact lens designs. Spectacles should be the least invasive and most accessible method for slowing myopia progression [15].

Spectacle lenses with defocus technology specifically designed for myopia progression control have been recently introduced in clinical practice because of their convenience, safety, and relatively low cost [12,14,23]. One of these kinds is the Defocus Incorporated Multiple Segments (DIMSs) lens, which has emerged as a promising treatment for myopia control [16]. This dual-focus lens consists of a central optical zone for correcting distance refractive error, and an annular mid peripheral focal zone with multiple segments having a relative positive power of +3.50 Ds [16]. This optical design, which provides a myopic defocus in the peripheral retina while maintaining clear central vision, is hypothesized to modulate ocular growth and mitigate the elongation of the eyeball, a key factor in myopia progression. Several clinical studies, mainly focused on the Asiatic population, have evaluated the effectiveness of defocus lenses in slowing myopia progression, with encouraging results [12,15].

Several mechanisms underlie the efficacy of defocus lenses in myopia control. Myopic defocus in the peripheral retina is believed to inhibit axial elongation by stimulating the release of retinal neurotransmitters associated with scleral remodeling. Moreover, defocus lenses may influence visual feedback mechanisms, such as accommodation and vergence, which play a role in regulating ocular growth. By providing clear central vision and myopic defocus in the periphery, these lenses may help to maintain optical balance and signal retinal homeostasis.

Recently [1,3,14], the potential synergistic effects of combining defocus lenses with other myopia control strategies, such as atropine therapy or outdoor activities, have been explored. Combining defocus lenses with low-dose atropine has shown promising results in further reducing myopia progression compared to either intervention alone [3]. Similarly, interesting findings have been reported regarding the beneficial effects of outdoor activities in conjunction with defocus lenses.

However, age-specific results for DIMS spectacle lenses are still not widely available in the literature. The existing studies suggest that DIMS spectacles represent a promising intervention for myopia control in children and adolescents. Further research specifically examining the age-related effects of DIMS spectacles on myopia progression could provide valuable insights into the optimal use of this treatment across different age groups.

The aim of this study is to evaluate the effectiveness of DIMS spectacle lenses in myopia progression control as a function of age in European pediatric patients.

## 2. Methods

This was a non-randomized experimenter-masked retrospective controlled observational study of European children with documented progressive myopia. The inclusion criteria were patients between 6 and 16 years of age, progressive myopia with cycloplegic spherical equivalent from −0.50 to −4.00 Ds, refractive astigmatism less than 2.0 Ds, and anisometropia under 1.0 Ds. The exclusion criteria were suspected genetic syndromes (e.g., Stickler, Marfan, etc.) and all systemic or eye diseases (such as glaucoma, congenital or juvenile cataract, retinal diseases, or any form of strabismus). These criteria were used to select participants for the study and ensure that the sample population met specific requirements related to age, ethnicity, refractive error, and ocular health status. All participants underwent a full ophthalmological assessment including symptoms and history, orthoptic testing, refraction (with cycloplegic autorefraction), and dilated fundoscopy.

Participants who met the inclusion criteria and did not meet any of the exclusion criteria were considered to be eligible in this retrospective study on myopia progression control intervention. Participants were allocated to receive DIMS (Hoya^®^ MiyoSmart®, Hoya Miyosmart, Tokyo, Japan) spectacle lenses or wear single-vision spectacle lenses. All the pediatric patients considered in the treatment groups wore the DIMS spectacle lenses, while subjects of the control groups wore ordinary single-vision spectacle lenses. The period considered for enrolment lasted from the 1 December 2020 to the 30 June 2021.

Informed consent was obtained for each patient, and all investigations followed the guidelines required by the institution. The study adhered to the Tenets of the Declaration of Helsinki (ClinicalTrials.gov: n. NCT06556849). 

At baseline and at 12-, 24-, and 36-month follow-ups, spherical equivalent refractive error (SE) under cycloplegia and axial length (AL) was measured for each patient. Myopia progression was calculated as the difference between mean SE and AL along the follow-up time. Cycloplegic refraction was measured with the PRK-5000 POTEK autorefractor (Potec, Daejeon, Republic of Korea), and the AL was calculated with the Nidek optical biometer AL-Scan (Nidek Co., Ltd., Gamagori, Japan). An average of five measurements of autorefraction and biometry for each eye were obtained for analysis. 

### Statistical Analysis

Baseline and follow-up data and the changes in SE and AL are presented as mean ± SD. Student’s independent *t*-test was used to compare myopia progression between DIMS spectacle lens wearer and control group patients, respectively, for age > 10 (groups A and B) and <10 (groups C and D). Changes in SE and AL at each time point were calculated and compared by a repeated-measures ANOVA and post hoc pairwise comparisons using Bonferroni corrections. Linear regression analysis was calculated to evaluate SE and AL change as a function of patient age for each group. A *p* value of less than 0.05 was considered significant.

## 3. Results

A total of 80 participants with 36-month follow-ups were included in the study. No treatment-related adverse event was reported. To determine the age-specific myopia progression, individuals were further categorized into a group aged 10 years or younger and a group over 10 years. In the attempt to evaluate DIMS spectacle lens effectiveness as a function of age, the results were stratified into four groups: patients wearing DIMS spectacle lenses older or younger than 10 years (group A, 20 patients mean age 13.6 ± 2.2, and group C, 20 patients mean age 9.0 ± 1.2) and age-matched control groups (group B, 18 patients mean age 13.2 ± 2.5, and group D, 22 patients mean age 8.5 ± 0.9) wearing single-vision spectacle lenses. 

**Figure 1:**
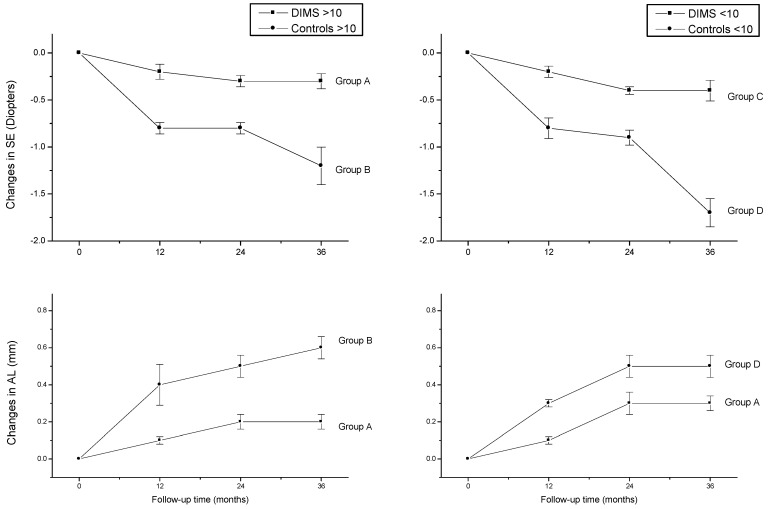
Changes in spherical equivalent refraction (SE) and axial length (AL) from baseline to 36 months in DIMS (A and C) and control (B and D) groups.

In total, 38 were over 10 years (group A and B) and 42 were 10 years or younger (group C and D). At baseline (Table 1), no significant differences in mean age and SE and AL value among corresponding age groups were observed (*p* > 0.05). Table 2 summarizes the results recorded at the 12-, 24-, and 36-month follow-ups. In the DIMS groups, the SE mean values did not change significantly over time (repeated-measures ANOVA, *p* > 0.05), whereas a statistically significant difference was found in the AL mean values between baseline and second year time points only for group C. Mean SE and AL changes were significantly different (*p* < 0.05) between the DIMS and control groups at each time point of the follow-ups. Post hoc analyses indicated that SE progression significantly increased (*p* < 0.05) in the third year in respect to first and second year mean values in the control group, whereas no significant variations were observed in the DIMS group along the follow-up times. A statistically significant difference (*p* < 0.05) was found in the AL changes between baseline and second year mean values in groups C and D; a similar result has been found in group A, whereas variations were statistically significant between the database and first year time points in group B (Figure 1). 

**Figure 2:**
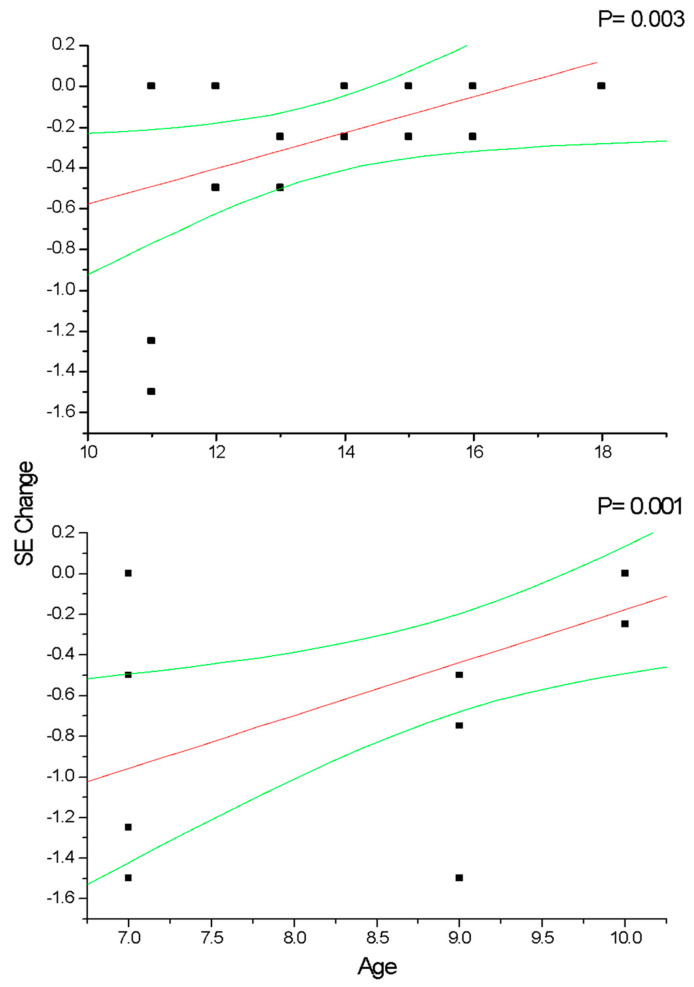
This figure shows the correlation between patient age and cycloplegic spherical equivalent (SE) change in myopic patients older (group A) and younger (group C) than 10 years of age that wore DIMS lenses. In both groups, the SE significantly decreases (*p* < 0.05) according to the patients’ age increases. Linear regression and 95% confidence limits are shown.

**Figure 3:**
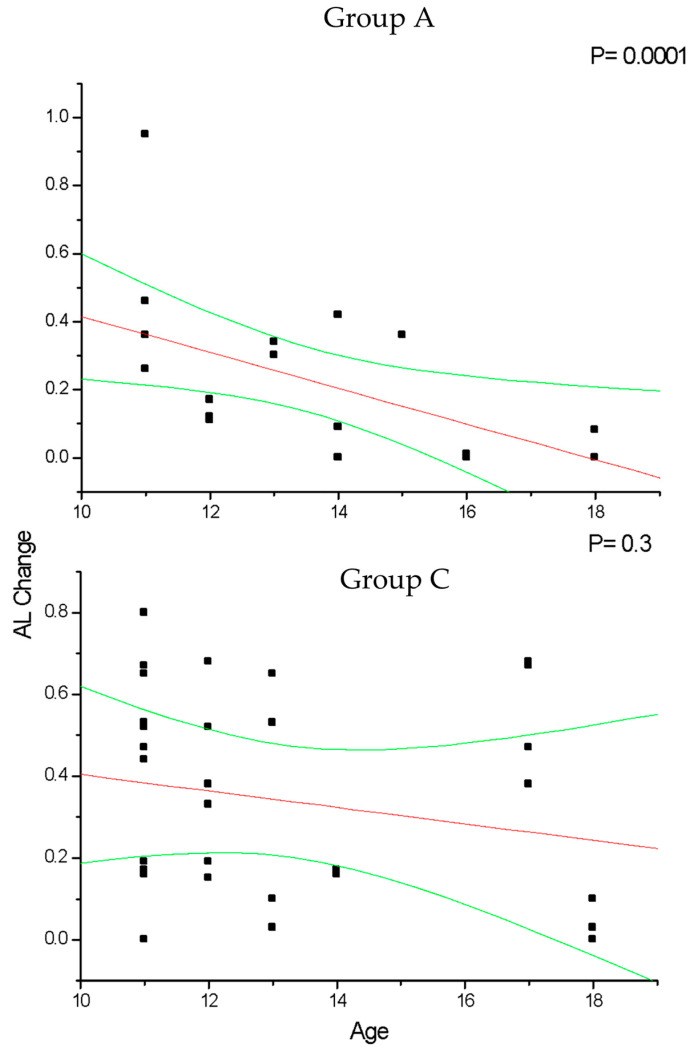
This figure shows the significant correlation between patient age and axial length (AL) change in myopic patients that wore DIMS lenses older than 10 years (group A) but not in younger ones (group C). In group A, AL increase significantly decreases according to the patients’ age increases. Linear regression and 95% confidence limits are shown.

**Figure 4:**
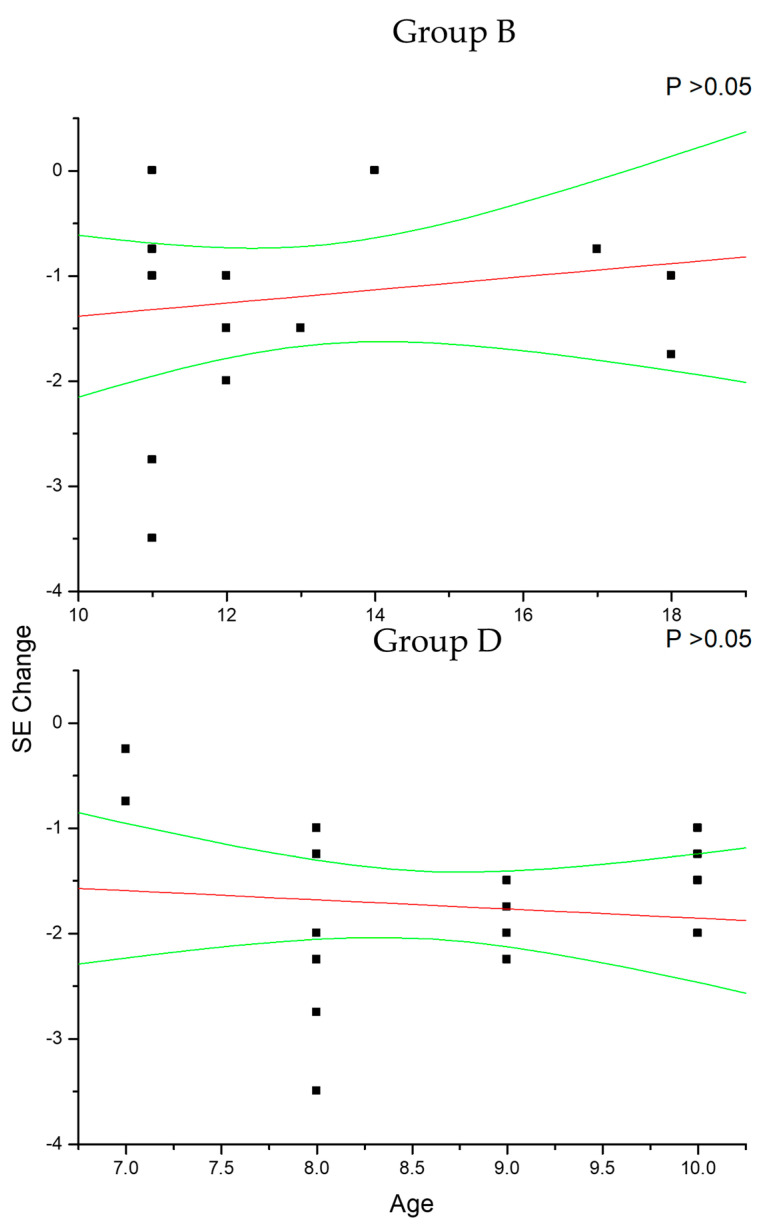
This figure shows the non-significant correlation between patient age and cycloplegic spherical equivalent (SE) change in control myopic patients older (group B) and younger (group D) than 10 years of age. Linear regression and 95% confidence limits are shown.

**Figure 5:**
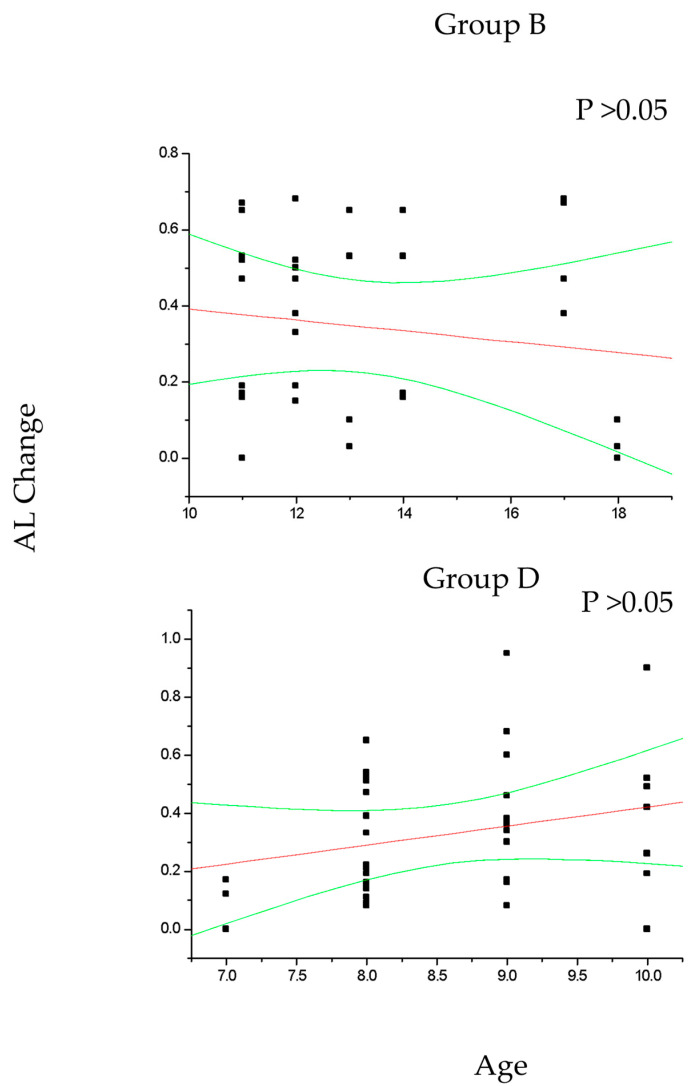
The figure shows the non-significant correlation between patient age and axial length (AL) change in control myopic patients older (group B) and younger (group D) than 10 years of age. Linear regression and 95% confidence limits are shown.

In group A and C (Figure 2), the change in SE recorded at the 36-month follow-up results positively correlated with patient age (*p* < 0.05), which means that increasing age was correlated with decreasing myopia progression. Differently, the change in AL (Figure 3) recorded at the 36-month follow-up appears inversely correlated with age in group A (*p* < 0.05) but not in group C (*p* > 0.05), which means that the increasing age was correlated with decreasing myopia progression only for DIMS spectacle lens wearers older than 10 years of age. Differently, control groups B and D did not show any significant linear correlation between SE (Figure 4) and AL (Figure 5) change and patients’ age (*p* > 0.05). 

## 4. Discussion

Myopia is a complex condition influenced by various factors such as genetics, environmental exposures, and lifestyle habits [1,13,14]. Few papers reported epidemiologic data on European myopic children, particularly with respect to myopia progression. 

Tricard et al. [24] published a prospective study involving a nationwide cohort in order to describe the progression of myopia in France using a cohort of individuals aged 4–17 years at baseline and followed for up to 6.5 years from 2013 to 2019. The authors recorded that factors associated with faster myopia progression were gender, with girls being more prone to progression than boys, higher myopia at baseline, and age between 7 and 12 years. Multivariate analysis showed that younger individuals aged 4–12 years, girls, and individuals with higher myopia at baseline were more likely to develop high myopia. The authors found that age is the most important factor in determining the mean progression rate and the relative speed, but they observed that age is not a monotonic factor, with 7–9-year-old myopes progressing faster and both younger and older children progressing more slowly. The slower progression in younger children, with very young onset of myopia, could reflect a different etiology. Ethnicity represents an important factor for determining a different rate of myopia progression; in fact, Asian children showed a faster progression than European children [5].

Tailored therapy could allow healthcare providers to customize treatment plans based on the specific needs and characteristics of each patient, optimizing the effectiveness of interventions [1,13,14]. Several experimental studies [25,26,27,28,29,30] demonstrated that myopic eye growth could be inhibited by inducing myopic defocus using dual focus lenses. The efficacy of DIMS lenses in slowing myopia progression has been largely reported, but up until now, few authors have analyzed the relationship between myopic progression (in terms of SE and AL change) and patient age.

Age-specific results for the efficacy of DIMS spectacle lenses in slowing myopia progression are important for several reasons. Different age groups may respond differently to myopia control interventions. Age-specific results can help to identify the most effective age range for using DIMS spectacle lenses, allowing for tailored treatment approaches based on age-related factors. Understanding how the efficacy of DIMS lenses varies as a function of age could also help to determine the optimal timing for initiating treatment. Age-specific results can guide healthcare providers in recommending the most appropriate age to start the use of DIMS spectacles in the attempt to achieve maximum effectiveness. Age-specific data can provide insights into the long-term outcomes of using DIMS spectacles for myopia control. By analyzing how myopia progression changes over time depending on age, researchers and clinicians can better predict the sustained benefits of treatment. Age-specific results could finally inform the development of targeted treatment strategies for specific age groups. This can include adjusted treatment protocols, follow-up schedules, and intervention combinations based also on the age-related responses to DIMS spectacle lenses.

Lam et al. [16] performed a 2-year double-masked randomized controlled trial in Chinese children aged 8–13 years, with myopia between −1.00 and −5.00 Ds and astigmatism ≤ 1.50 Ds, and reported that age was the only significant parameter which affects the efficacy of DIMS spectacle lenses in slowing myopia progression. The authors observed that the effect of myopia control with DIMS lenses was greater in children older than 10 years of age. In fact, 80% of DIMS spectacle lens wearers who showed myopia progression were children aged 8–9 years. The authors observed that these findings could be related to different retinal profiles or peripheral refraction among children. In the case of a high amount of peripheral hyperopia, the value of effective myopic defocus at the peripheral retina will be less, thereby minimizing the treatment effect. Because younger children presented a myopic peripheral refraction at baseline, and because the effectiveness of DIMS spectacle lenses depends on the counterbalance between the hyperopic defocus of the eye and the myopic defocus achieved by the DIMS lens, when a baseline peripheral myopic refraction is combined with the myopic defocus induced by the lens, the total amount of myopic defocus at the mid-periphery retina could be too much to provide a satisfactory control of myopia progression.

Long et al. [31] in a retrospective study compared Chinese myopic patients aged 6 to 15 years with SE refraction ranging between −0.50 and −8 Ds that wore DIMS and single-vision spectacle lenses, and, in accordance with Lam et al. [12], they found that the chances of achieving myopia control with DIMS spectacle lens design were better in children aged 10 to 15 years than in children aged 6 to 9 years. Previous papers on this topic are summarized in Table 3.

To our knowledge, this is the first study that evaluates DIMS technology effectiveness as a function of age in European pediatric patients. In fact, Lam et al. [12] and Long et al. [31] limited their study to Chinese children, and no author up until now has evaluated DIMS spectacle lenses’ effectiveness in other ethnic populations.

Some authors evaluated the effectiveness of DIMS lenses in the European population. Recently, Nucci et al. [3] compared the efficacy of the DIMS spectacle lens, atropine eye drops, and a combination of DIMSs and atropine in slowing the progression of myopia in European patients. These results showed that both DIMS lenses and 0.01% atropine were individually effective in slowing myopia progression in this population. However, the study did not provide detailed results based on different age groups, while the overall findings indicated that both DIMS spectacles and atropine were effective in reducing myopia progression in the population of European children and adolescents studied. The combination of atropine and DIMSs was shown to be particularly effective in slowing myopia progression compared to either treatment alone.

A recent review of a myopia control study reported that age does not affect the effectiveness of treatment options [32]. However, some authors [12,31] also found that in the Chinese pediatric population who had worn DIMS spectacle lenses, older children showed a slower myopia progression, while 8-year-old patients showed more myopia progression and AL elongation; our findings confirm that in European pediatric patients, DIMS technology does not produce any adverse effect and slows myopia progression. Our data suggest that DIMSs’ effectiveness, evaluated as a function of age, significantly improves in patients aged > 10 years. In fact, while SE change results were significantly correlated in group A as well as in C, AL increase was inversely correlated only for patients older than 10.

These findings appear in accordance with several authors that have already observed that the primary endpoints for judging efficacy in clinical trials of myopia control intervention should include change in axial length more than in refractive error [15]. 

In this study, there were some limitations. The parents generally chose for their own child which kind of spectacles to wear, and, because of various reasons, we were not able to perform a 6-month monitoring. Moreover, the non-randomized retrospective design and the limited number of patients enrolled mean that more extensive studies are required.

The relationship broadly reported between age of onset and severity of myopia in late childhood confirms the importance of screening children at high risk of early-onset myopia in order to start early treatment. Genetics have supported the significant association between parental myopia and onset of childhood myopia, and environmental exposures including education, near work, and outdoor activities have been identified as key factors in the development of myopia [4]. Cross-linked studies for investigating possible correlations between myopia onset, possible related risk factors, and treatment options could give important future indications, improving the therapeutic approach.

To recap, myopia frequently appears in childhood, with a peak incidence occurring between 8 and 10 years of age. There is major disparity in the prevalence of myopia in children according to ethnic origin. The progression of myopia has been analyzed in various studies, and a younger age of myopia onset or longer duration of myopia progression are strong predictors of high myopia. Ethnicity is clearly an important factor in the rate of progression, with children of East Asian ancestry progressing faster than those of European ancestry [24]. 

Although emerging treatments for myopia are promising and some have been incorporated into clinical practice, identifying populations who require and benefit from intervention remains the most important step for optimizing clinical behavior.

## 5. Conclusions

In conclusion, age-specific results for the efficacy of DIMS spectacle lenses in myopia control are crucial for optimizing treatment outcomes, tailoring interventions to specific age groups, and guiding clinical decision making in the management of myopia in children and adolescents because early management seems crucial to mitigating the long-term consequences on ocular health.

Further randomized prospective studies with a higher number of patients are needed to validate these early findings; however, our retrospective study suggests the effectiveness of DIMS spectacle lenses in slowing myopia progression and axial elongation among European children with progressive myopia, mainly if older than 10 years of age. 

## Figures and Tables

**Table 1:** Participant characteristics at baseline.

	TOTAL	DIMS	CONTROL	*p*
Patients number	80	40	40	*p* > 0.05
Age (years)		11.5 ± 3	11.2 ± 2.9	*p* > 0.05
SE ^ (diopters)		−2.9 ± 2	−2.7 ± 2.4	*p* > 0.05
AL * (mm)		24.3 ± 1.1	24.4 ± 0.9	*p* > 0.05

DIMS: Defocus Incorporated Multiple Segment (DIMS) spectacles; ^ SE: cycloplegic spherical equivalent; * AL: axial length.

**Table 2:** Mean changes in the cycloplegic spherical equivalent (SE) and axial length (AL) from baseline to 12, 24, and 36 months in the DIMS and control groups.

Time (Months)	Patients Age (Years)	DIMS (SE ^, Diopters)	CONTROL (SE ^, Diopters)	DIMS (AL *, mm)	CONTROL (AL *, mm)
12	<10	−0.2 ± 0.3 **	−0.8 ± 0.5	0.1 ± 0.1 **	0.3 ± 0.1
12	>10	−0.2 ± 0.4 **	−0.8 ± 0.3	0.1 ± 0.1 **	0.4 ± 0.4
24	<10	−0.4 ± 0.2 **	−0.9 ± 0.4	0.3 ± 0.3 **	0.5 ± 0.3
24	>10	−0.3 ± 0.3 **	−0.8 ± 0.3	0.2 ± 0.2 **	0.5 ± 0.3
36	<10	−0.4 ± 0.5 **	−1.7 ± 0.7	0.3 ± 0.2 **	0.5 ± 0.3
36	>10	−0.3 ± 0.4 **	−1.2 ± 0.9	0.2 ± 0.2 **	0.6 ± 0.3

DIMS: Defocus Incorporated Multiple Segment (DIMS) spectacles; ^ SE: cycloplegic spherical equivalent; * AL: axial length; **: *p* < 0.05.

**Table 3:** Effectiveness of Defocus Incorporated Multiple Segment (DIMS) spectacles in slowing myopia progression reported in previous studies.

AUTHORS	DURATION (Years)	PROGRESSION SE (Ds) ^	PROGRESSION AL (mm) *
Long et al. (2023) [31]	1	−0.51 ± 0.50	NOT REPORTED
Lam et al. (2020) [16]	2	−0.38 ± 0.06	0.21 ± 0.02
Lam et al. (2023) [12]	6	−0.40 ± 0.72	0.28 ± 0.28

^ SE: cycloplegic spherical equivalent (diopters); * AL: axial length (millimeters).

## Data Availability

The data presented in this study are available on request from the corresponding author.
